# A Pediatric Case of Glioblastoma Multiforme Associated With a Novel Germline p.His112CysfsTer9 Mutation in the *MLH1* Gene Accompanied by a p.Arg283Cys Mutation in the *TP53* Gene: A Case Report

**DOI:** 10.3389/fgene.2019.00952

**Published:** 2019-10-22

**Authors:** Aleksandra Stajkovska, Sanja Mehandziska, Rodney Rosalia, Margarita Stavrevska, Marija Janevska, Martina Markovska, Ivan Kungulovski, Zan Mitrev, Goran Kungulovski

**Affiliations:** ^1^Sector of Genetics, Bio Engineering LLC, Skopje, Macedonia; ^2^Laboratory of Genetics and Personalized Medicine, Zan Mitrev Clinic, Skopje, Macedonia

**Keywords:** next-generation sequencing, North Macedonia, hereditary cancer syndromes, Lynch syndrome, *TP53*, *MLH1*, Li-Fraumeni, case report

## Abstract

Targeted gene panel testing has the power to interrogate hundreds of genes and evaluate the genetic risk for many types of hereditary cancers simultaneously. We screened a 13-year-old male patient diagnosed with glioblastoma multiforme with the aim to get further insights into the biology of his condition. Herein, we applied gene panel sequencing and identified a heterozygous frameshift mutation c.333_334delTC; p.His112CysfsTer9 in the *MLH1* gene in blood and tumor tissue accompanied by a known heterozygous missense variant of unknown significance c.847C > T; p.Arg283Cys in the *TP53* gene. Parental screening revealed the presence of the same *TP53* variant in the father and the same *MLH1* variant in the mother, who was in fact undergoing treatment for early-stage breast cancer at the time of her son’s unfortunate diagnosis. This case reports for the first time the co-occurrence of a genetic mutation in the *MLH1* gene of the mismatch repair pathway, commonly associated with the Lynch syndrome, accompanied by a rare variant in the *TP53* gene. This report underlines the need for broad panel gene testing in lieu of single-gene or syndrome-focused gene screening and evaluation of the effects of multiple pathogenic or modifier variants on the phenotypic spectrum of the disease.

## Background

### MMR-Dependent Hereditary Cancer Syndromes

The DNA mismatch repair (MMR) machinery is a highly conserved cell-intrinsic fail-safe system that recognises and repairs mismatched bases emanating from spurious DNA replication, recombination, or chemical/physical insults (Richman, 2015). A malfunction of the MMR machinery may lead to microsatellite instability, which in turn increases the rate of mutations.

By virtue of this, germline genetic mutations in the *MLH1*, *MSH2* (or through *EPCAM*), *MSH6*, and *PMS2* genes lead to an inborn functional deficiency of the MMR pathway, thereby significantly increasing the risk of cancer. Mutations in the MMR pathway are associated with hereditary cancer syndromes such as Lynch syndrome, Turcot syndrome, and Muir–Torre syndrome.

Turcot syndrome (OMIM 276300) is a disease that manifests *via* multiple adenomatous colon polyps; patients have an increased risk of colorectal cancer and brain cancers, namely glioblastoma. Turcot syndrome typically follows an autosomal dominant inheritance pattern. It is closely associated with other rare hereditary cancers, such as familial adenomatous polyposis or Lynch syndrome (Hegde et al., 2014; Khattab and Monga, 2019).

Genetic mutations in *APC* gene associated with familial adenomatous polyposis, or a mutation in one of the MMR genes, the *MLH* gene in particular associated with Lynch syndrome, form the molecular basis for most cases of Turcot syndrome (Carethers and Stoffel, 2015). There is a dichotomous trend observed in regard to the etiology and clinical presentation of the hereditary brain cancers; *APC* mutations typically trigger an oncogenic pathway leading to medulloblastoma. In contrast, mutations in the MMR machinery usually lead to glioblastoma multiforme (GBM) (Alifieris and Trafalis, 2015), a devastating brain cancer; diagnosis of GBM is associated with a dire clinical outcome in the majority of cases. Despite aggressive combinatorial therapy, survival of (adults) ranges between 8 and 18 months, depending on the extent of the disease (Kohlmann and Gruber, 1993; Sehgal et al., 2014; Stepanenko and Chekhonin, 2018).

### *TP53*-Dependent Hereditary Cancer Syndrome

*TP53* is a tumor-suppressor gene, encoding the p53 protein, which has a crucial role in the regulation of cell proliferation (Wawryk-Gawda et al., 2014). In particular, p53 regulates apoptosis, genomic stability, and angiogenesis (Pentimalli, 2018). Li-Fraumeni syndrome (LFS) (OMIM 151623) is a rare disorder, inherited in an autosomal dominant manner, caused by germline mutations in the *TP53* gene. Mutations that lead to suboptimal function or total loss of function of the p53 lead to compromised tumor suppression and cell proliferation. Consequently, individuals with dysfunctional p53 are highly susceptible to a broad range of cancers (Olivier et al., 2010).

The tumors most closely associated with LFS are so-called “core” cancers; brain cancers form part of this group of LFS-associated malignancies (Malkin, 2011; Sorrell et al., 2013; McBride et al., 2014; Kratz et al., 2017). The Chompret criteria have been proposed for the screening of patients suspected for LFS (Tinat et al., 2009).

Individuals with LFS are eligible for treatment; a personalized approach is required according to the intrinsic properties of the tumor. In addition, caution is warranted due to the known adverse effects of conventional radiotherapy. Several (pre-)clinical studies have shown an increased risk for radiation-induced cancers in LFS patients.

## Case Presentation

**Figure 1:**
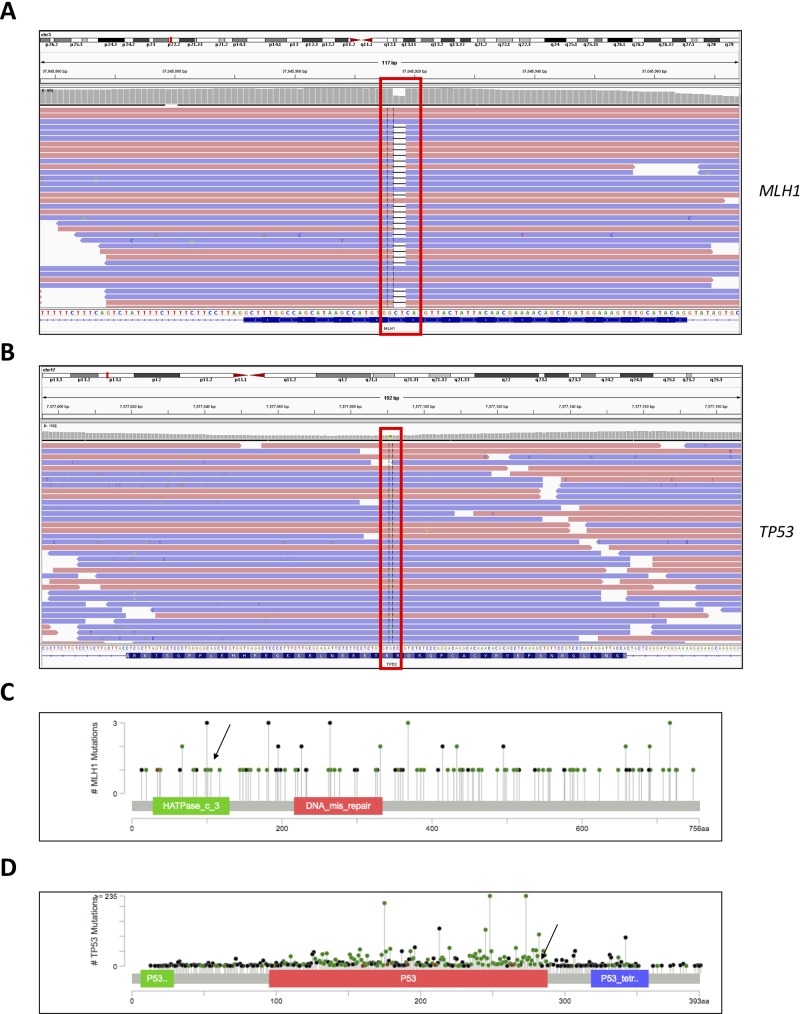
Genome browser views of the **(A)** *MLH1* gene showing sequence alignment around the region of the detected c.333_334delTC mutation and **(B)** *TP53* gene showing sequence alignment around the region of the detected c.847C > T mutation. **(C)** Lollipop scheme of the *MLH*1 gene, pinpointing the region of the detected mutation. **(D)** Lollipop scheme of the *TP53* gene, pinpointing the region of the detected mutation.

The proband was a 13-year-old boy, who was referred for genetic testing due to a suspected hereditary cancer syndrome, following a diagnosis of GBM WHO grade IV. He previously underwent surgical resection combined with adjuvant temozolomide chemotherapy. The father reported no family history of cancer; however, the mother was diagnosed with breast cancer at the age of 56 and underwent a bilateral mastectomy. Furthermore, the maternal grandmother was also diagnosed with breast cancer at old age. Upon taking written and signed informed consent from the proband’s legal guardians, gene panel sequencing revealed a novel heterozygous frameshift mutation c.333_334delTC; p.His112CysfsTer9 in the *MLH1* gene and another known, but rare, heterozygous missense VUS c.847C > T; p.Arg283Cys in the *TP53* gene (**Table 1**; **Figure 1**).

**Table 1:** Properties of detected variants, symptoms and medical history.

Method	*Gene*	Nucleotide change	Protein change	rsID	MAF	Clinvar sig	ACMG	Novelty	Inheritance	Symptoms and family history
TruSight Cancer; MMR sequencing; Sanger sequencing	*MLH1*	c.333_334delTC	p.His112CysfsTer9	/	/	/	PVS1, PM1, PM2	Unknown	Dominant	**Proband**: Glioblastoma multiforme, age 12 **Mother**: breast cancer, age 56, and bilateral mastectomy **Grandmother**: breast cancer
	*TP53*	c.847C > T	p.Arg283Cys	rs149633775	0.00008	Uncertain significance	PM1, PP3, PP5	Known VUS	Dominant	

The mutation in the *MLH1* gene results in a truncated protein and most likely leads to loss of function, predisposing carriers to hereditary malignant syndromes, for example Turcot syndrome or the related Lynch syndrome. The discovered variant in the *TP53* gene meets PM1, PP3, and PP5 ACMG pathogenicity criteria (Richards et al., 2015); in the ClinVar database, it is annotated as a *variant of uncertain significance—*albeit with probable functional relevance. The presence of the *MLH1* mutation was validated independently in blood and tumor tissue with next-generation sequencing (NGS) (130 brain tumor-relevant genes) and Sanger sequencing. In addition, DNA methylation analysis of the tumor tissue in comparison with a reference database of 2,800 tumors, categorized the tumor in the methylation class glioblastoma, isocitrate dehydrogenase wild type, subtype RTK III. These data indicated *MGMT* promoter methylation and potential loss of *CDKN2A*.

**Figure 2:**
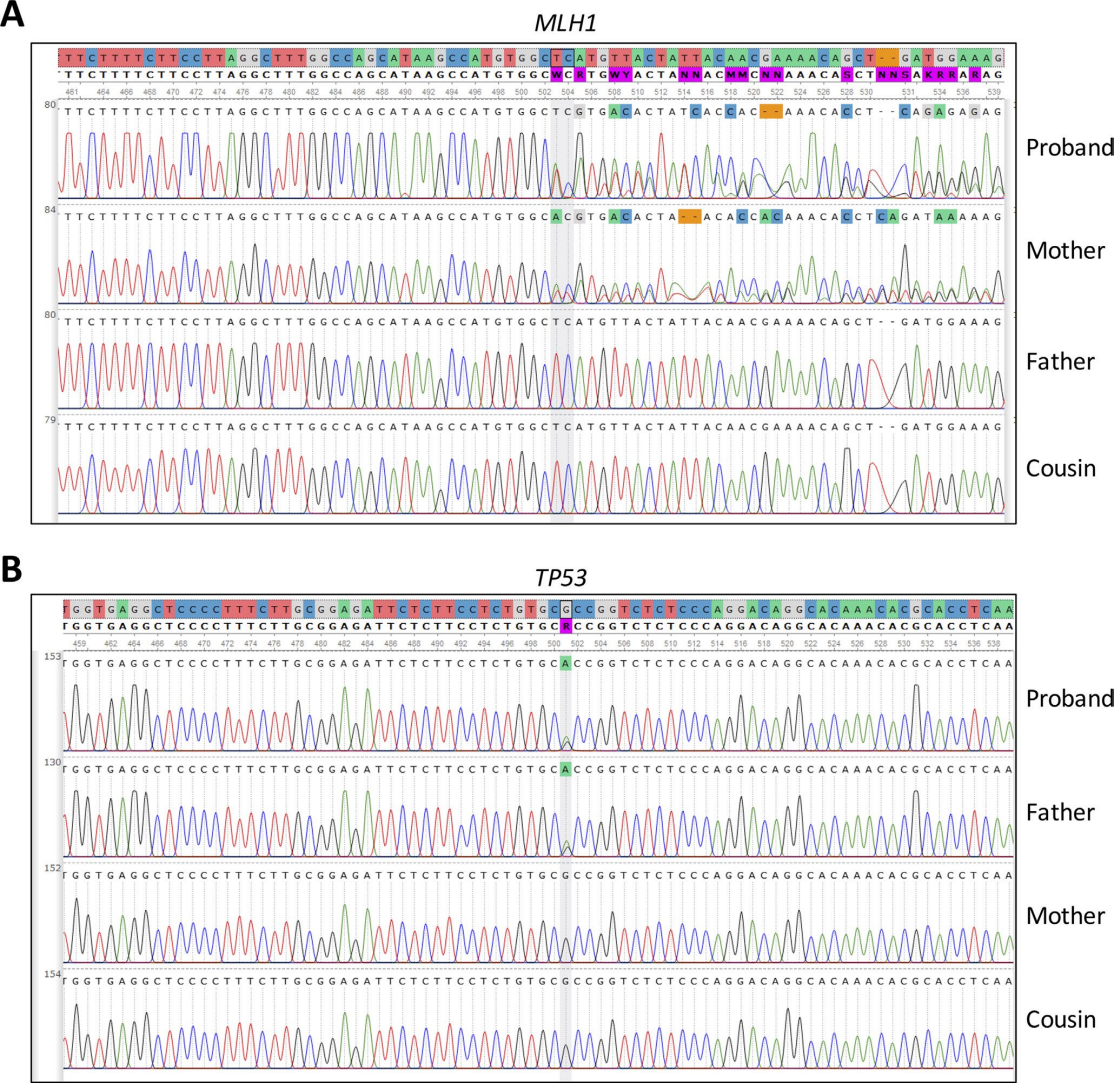
Sanger sequencing of **(A)** *MLH*1 gene, pinpointing the presence or absence of the c.333_334delTC mutation in the patient and family members and **(B)** *TP53* gene, pinpointing the presence or absence of the c.847C > T mutation detected by in the patient and family members.

Moreover, we evaluated the NGS result and inheritance pattern by Sanger sequencing—we concluded that the *TP53* mutation was inherited from the paternal side, while the *MLH1* mutation was inherited from the maternal side. Sanger sequencing of a maternal cousin failed to detect the *TP53* and *MLH1* mutations (**Figure 2**).

### Laboratory Tests

The patient and his family underwent gene panel sequencing (TruSight Cancer, Illumina) or Sanger sequencing, respectively. In brief, DNA was extracted from 400 µl of whole blood on a SaMag-12 automatic nucleic acid extraction system (Sacace Biotechnologies, Como, Italy). Libraries were prepared following the manufacturer’s recommendations, and raw sequences were obtained from the NextSeq machine (Illumina, San Diego, USA). Sequence quality control, single nucleotide polymorphism, and insertion/deletion calling, together with advanced variant annotation were done with proprietary technologies such as the Sophia DDM platform (Sophia Genetics, Saint-Sulpice, Switzerland). The NGS panel of brain-tumor-relevant genes, the DNA methylation analysis with the 850K Illumina array, and methylation classification (internal classifier V11b2) were carried out at the University Clinic in Heidelberg.

## Discussion and Concluding Remarks

In this case report, we used gene panel sequencing to evaluate the hereditary cancer risk of a pediatric GBM patient and his family and to get an insight into the biology of the tumor.

Through this approach, we identified two heterozygous highly probable disease-causing mutations: c.333_334delTC; p.His112CysfsTer9 in the *MLH1* gene, and c.847C > T; p.Arg283Cys in the *TP53* gene. The proband inherited the pathogenic variant in the *MLH1* gene from his mother. We hypothesize that the frameshift mutation in the *MLH1* gene is most likely the primary oncogenic driver and the main culprit causing cancer proclivity in this pediatric case of GBM.

Given the rare clinical presentation and absence of abdominal symptoms, the patient was never suspected of Turcot syndrome or Lynch syndrome. Hence, colonoscopy screening was never performed. We are unable to exclude the presence of other primary (pre-)malignant lesions at distal sites, which could have strengthened the diagnosis. However, the hereditary genetic profile strongly points to the aforementioned cancer syndromes manifesting as GBM.

GBM (Alifieris and Trafalis, 2015) is an epithelial tumor of the central nervous system with frequent genetic and epigenetic alterations (Heiland et al., 2017) and a worldwide incidence of <10% that commonly manifests as a solitary lesion; multiple GBM lesions are rare. GBM manifests in adults between the age of 45 and 70 years old (Zhang et al., 2016). Conversely, our patient developed aggressive intracranial malignancy at a very young age, prompting the suspicion of multiple oncogenic or modifier mutations. Indeed, further analysis uncovered the paternally inherited mutation, c.847C > T; p.Arg283Cys, in the *TP53* gene.

We speculate that the p.Arg283Cys variant in *TP53* served as an additional oncogenic driver or modifier, resulting in the unusually early onset of GBM. There are several lines of evidence supporting this claim. First, Monti et al. showed that the p.Arg283Cys variant, among other *TP53* germline variants, showed severe deficiency to transactivate *MDM2*, *BAX*, and *PUMA*, but not *CDKN1A*, in a luciferase-based quantitative assay in yeast, when compared to the wild-type allele (Monti et al., 2011). Similarly, another *in vitro* functional study indicated that the germ-line p.Arg283Cys variant could still transactivate the *CDKN1A* but not the *BAX* gene and thus retained the ability to induce growth arrest of human glioblastoma cells (Fulci et al., 2002). Furthermore, it has been shown that the p.Arg283Cys p53 protein is cold sensitive and unable to activate p53-RE placed upstream of the *ADE2* reporter in yeast (Jagosova et al., 2012). These studies illustrate that the c.847C > T; p.Arg283Cys mutation, unlike other highly pathogenic mutations in the *TP53* gene (Pavletich et al., 1993; Muller and Vousden, 2014; Shajani-Yi et al., 2018), causes a partial loss of function with unclear clinical repercussions.

Second, in genetic studies, the p.Arg283Cys mutation was identified together with a nonsense variant in *BRCA2* in a patient with metachronous breast cancers and a subsequent leiomyosarcoma, with a family history of ovarian cancer, breast and ovarian cancer, and glioblastoma (Manoukian et al., 2007). In addition, the c.847C > T; p.Arg283Cys mutation was the only variant detected in a *CDH1* negative gastric cancer patient, with a family history of gastric cancer, leukemia, and liver cancer (Yurgelun et al., 2015).

Collectively, our current observations and published reports provide circumstantial evidence for the functional relevance of the p.Arg283Cys, *TP53* variant, but further studies are necessary to substantiate this claim. Moreover, functional studies are warranted to evaluate the ramifications of the co-occurrence of *MLH1* loss-of-function mutations and the p.Arg283Cys mutation in *TP53*.

Targeted gene panel testing of known cancer-associated genes is a cost-effective diagnostic tool to simultaneously evaluate patients and their relatives suspected of hereditary malignant syndromes. As genetic testing is getting readily available to an increasing number of institutions, we anticipate that the number of similar cases will increase. This is a cautionary tale for clinicians, medical geneticists, and genetic counselors to take into account the possibility of a patient having two or more disease-causing or disease-modifying variants, which might influence the severity, tissue specificity, and onset of the disease (Cohen et al., 2016)

## Data Availability Statement

FASTQ data have been deposited to the NCBI under the accession number PRJNA516553 (https://www.ncbi.nlm.nih.gov/sra/PRJNA516553).

## Ethics Statement

Written and signed informed consent was obtained from all subjects or their legal guardians for participation and publication of this case study.

## Author Contributions

GK conceived and designed this case study. GK processed, analyzed, and interpreted the sequencing data with the help of AS, SM, MS, MM, and MJ. GK, AS, and SM contributed to genetic counseling. RR, IK, and ZM contributed to the recruitment of patients in the hospital and contributed intellectually. GK and RR wrote the manuscript. AS and SM contributed equally to the manuscript. All authors contributed to the improvement of the manuscript and read the final version of the manuscript.

## Funding

The authors declare that this study has not received any funding. It was carried out as part of the routine clinical work at the Zan Mitrev Clinic.

## Conflict of Interest

Author AS, MJ,MM,IK AND GK were employed by company Bio Engineering LLC. Author SM, RR, MS, ZM were employed by company Zan Mitrev Clinic.
